# Docosahexaenoic Acid Esters of Hydroxy Fatty Acid Is a Novel Activator of NRF2

**DOI:** 10.3390/ijms22147598

**Published:** 2021-07-15

**Authors:** Siddabasave Gowda B. Gowda, Takayuki Tsukui, Hirotoshi Fuda, Yusuke Minami, Divyavani Gowda, Hitoshi Chiba, Shu-Ping Hui

**Affiliations:** 1 Faculty of Health Sciences, Hokkaido University, Kita 12, Nishi 5, Kita-ku, Sapporo 060-0812, Japan; siddabasavegowda.bommegowda@hs.hokudai.ac.jp (S.G.B.G.); hirofuda@gmail.com (H.F.); minami.yusuke.t2@elms.hokudai.ac.jp (Y.M.); divyavani@hs.hokudai.ac.jp (D.G.); 2 Department of Nutrition, Sapporo University of Health Sciences, Nakanuma Nishi-4-3-1-15, Higashi-ku, Sapporo 007-0894, Japan; tsukui@sapporo-hokeniryou-u.ac.jp (T.T.); chiba-h@sapporo-hokeniryou-u.ac.jp (H.C.)

**Keywords:** FAHFAs, DHA, NRF2, lipid droplet, HRMS, LC/MS

## Abstract

Fatty acid esters of hydroxy fatty acids (FAHFAs) are a new class of endogenous lipids with interesting physiological functions in mammals. Despite their structural diversity and links with nuclear factor erythroid 2-related factor 2 (NRF2) biosynthesis, FAHFAs are less explored as NRF2 activators. Herein, we examined for the first time the synthetic docosahexaenoic acid esters of 12-hydroxy stearic acid (12-DHAHSA) or oleic acid (12-DHAHOA) against NRF2 activation in cultured human hepatoma-derived cells (C3A). The effect of DHA-derived FAHFAs on lipid metabolism was explored by the nontargeted lipidomic analysis using liquid chromatography-mass spectrometry. Furthermore, their action on lipid droplet (LD) oxidation was investigated by the fluorescence imaging technique. The DHA-derived FAHFAs showed less cytotoxicity compared to their native fatty acids and activated the NRF2 in a dose-dependent pattern. Treatment of 12-DHAHOA with C3A cells upregulated the cellular triacylglycerol levels by 17-fold compared to the untreated group. Fluorescence imaging analysis also revealed the suppression of the degree of LDs oxidation upon treatment with 12-DHAHSA. Overall, these results suggest that DHA-derived FAHFAs as novel and potent activators of NRF2 with plausible antioxidant function.

## 1. Introduction

Fatty acid esters of hydroxy fatty acids (FAHFAs) are a family of endogenous lipids composed of a fatty acid esterified with a hydroxy fatty acid at various positions. They are structurally diverse, with more than 51 families and 300 regioisomers identified [1]. FAHFAs are reported to exhibit important biological properties such as antidiabetic, anti-inflammatory, anti-apoptotic, and antioxidant activities [2,3,4]. Several in vitro and in vivo studies demonstrated the FAHFAs de novo synthesis in mammals by the transferring a fatty acid from a fatty acid-CoA to a hydroxy fatty acid via lipid acyltransferases [5]. They are also present in dietary plant sources as well as animal meats. In addition, FAHFAs are known to be the endogenous substrates for hydrolases such as ADTRP (androgen-dependent TFPI-regulating protein), AIG1 (androgen-induced gene 1 protein), and pancreatic lipase CEL (carboxy-ester lipase) [6,7]. The most studied FAHFAs are the palmitic-hydroxy stearic acid (PAHSA) with ester linkage at C_5_ to C_13_ [1,5,8]. PAHSAs augmented intestinal crypt Paneth cell bactericidal potency via GPR 120 and reduced the colonic T-cell activation and pro-inflammatory cytokine or chemokine expression [9]. Oral administration of 5/9-PAHSAs improved glucose tolerance and enhanced insulin sensitivity in insulin resistant mice on a high-fat diet [10].

Polyunsaturated fatty acid (PUFA) esterified FAHFA such as 13-docosahexaenoic acid hydroxy linoleic acid (13-DHAHLA) is also inhibited lipopolysaccharide-induced secretion of pro-inflammatory cytokines and stimulated the immune cells to limit macrophage activation during the inflammatory responses [11]. Importantly, dietary supplementation of docosahexaenoic acid (DHA) in diabetic patients increased the blood levels of DHAHLA and played a protective role as antidiabetic agents [11]. Hence, PUFAs derived FAHFAs are demonstrated to be more potent anti-inflammatory properties than saturated ones. To defend against oxidative stress induced cellular damages in various pathological conditions such as chronic kidney diseases, inflammatory bowel disease, pulmonary hypertension, and cardiovascular and neurodegenerative disorders, NRF2 activation is needed [12]. Hence, there is a growing need for the most promising NRF2 activators. The activated NRF2 is known to be involved in upregulation of antioxidant enzymes and plays a protective cellular function. Biosynthesis of FAHFAs can be augment oxidative stress via the NRF2- mediated antioxidant defense system [13]. Although the studies on antioxidant properties of FAHFAs are limited, our recent report demonstrated that eicosapentaenoic acid esterified 12-hydroxy stearic acid (12-EPAHSA) or oleic acid (12-EPAHOA) activated NRF2 signaling and also suppressed the oxidation of small lipid droplets (LDs) and oxidative stress induced by H_2_O_2_ [4]. Despite the numerous biological activities of PUFA-derived FAHFAs, a limited number of molecular species were explored. In this study, we aim to study the effect of novel synthetic docosahexaenoic acid esters of 12-hydroxy stearic acid (12-DHAHSA) or oleic acid (12-DHAHOA) on NRF2 activation, lipid metabolism, and LDs oxidation in human hepatocytes.

## 2. Results

**Figure 1:**
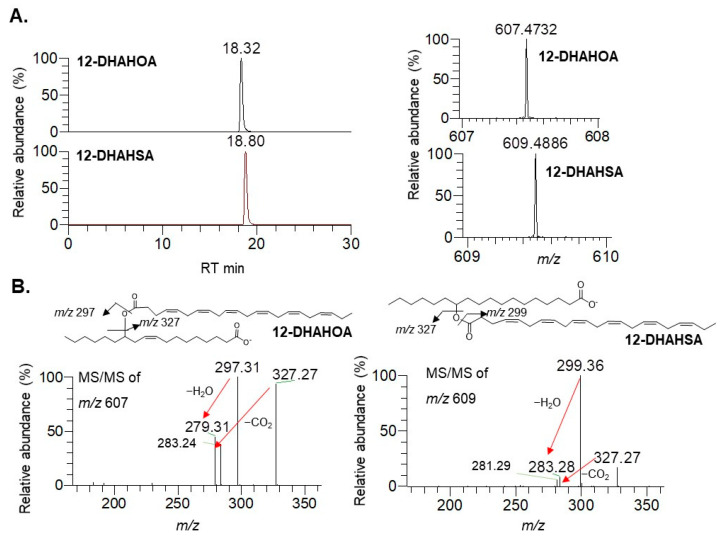
Structural characterization of DHA-derived FAHFAs. (**A**) Extracted ion chromatograms of 12-DHAHOA, 12-DHAHSA (left panel), and their respective high-resolution mass spectra (right panel). (**B**) MS/MS spectra of 12-DHAHOA and 12-DHAHSA.

The synthesis of docosahexaenoic acid (DHA) esters of 12-hydroxy stearic acid (12-HSA) and 12-hydroxy oleic acid (12-HOA) was performed by the method developed earlier in our laboratory for EPA-derived FAHFAs [4]. The column chromatographic purified compounds 12-DHAHOA and 12-DHAHSA were subjected to LC/MS analysis to confirm their structure. The extracted ion chromatograms, high-resolution masses, and MS/MS fragmentation pattern of 12-DHAHOA and 12-DHAHSA were provided in Figure 1. The compound 12-DHAHOA elutes at 18.3 min and is ionized in negative mode to produce [M-H]^−^ ion having the acquired mass 607.4732 (theoretical *m*/*z*: 607.4732, mass error: 0 ppm). The MS/MS spectra of 12-DHAHOA showed that the two major fragment ions at *m*/*z* 297 and *m*/*z* 327 correspond to their free fatty acids 12-HOA and DHA, respectively. 

The 12-HOA (*m*/*z* 297) loses a neutral molecule of water (18 Da) and produces a peak at *m*/*z* 279, whereas DHA (*m*/*z* 327) loses a neutral molecule of carbon dioxide (44 Da) to produce a peak at *m*/*z* 283, respectively. Hence, the observed MS and MS/MS patterns confirm the structure as 12-DHAHOA. Similarly, the compound 12-DHAHSA elutes at 18.8 min and is ionized in negative mode to produce [M-H]^−^ ion having acquired *m*/*z* 609.4886 (theoretical *m*/*z*: 609.4888, mass error: −0.32 ppm). The MS/MS spectra of 12-DHAHSA showed that the two product ions at *m*/*z* 299 and *m*/*z* 327 corresponds to 12-HSA and DHA, respectively. These fragment ions are further ionized and lose their neutral molecules of H_2_O and CO_2_ to produce *m*/*z* 281 and *m*/*z* 283 fragment ions, respectively. This type of saturated/polyunsaturated fatty acid-specific fragmentation is quite commonly observed in previous reports [14]. Overall, both the acquired MS and MS/MS fragmentation patterns confirm the structure as 12-DHAHSA.

**Figure 2:**
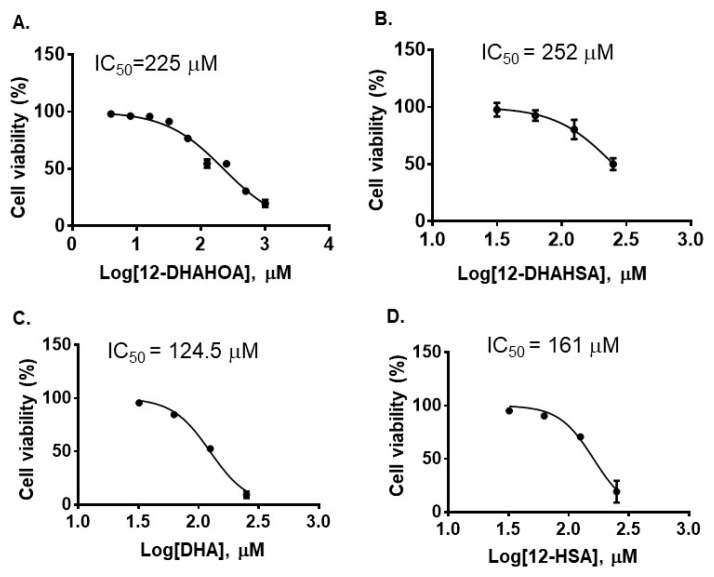
Cytotoxicity of DHA-derived FAHFAs and their free fatty acids. (**A**) 12-DHAHOA, (**B**) 12-DHAHSA, (**C**) DHA and (**D**) 12-HSA (mean ± SD (*n* = 6)).

**Figure 3:**
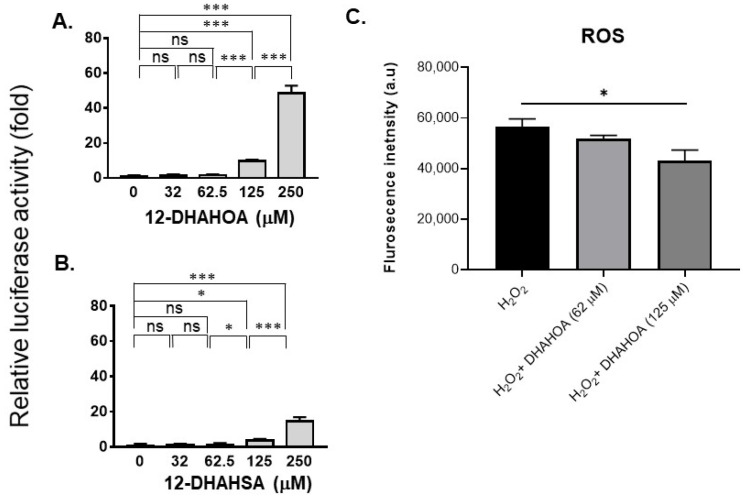
Evaluation of the antioxidant potential of DHA-derived FAHFAs in C3A cells. Reporter gene assay results of 12-DHAHOA (**A**) and 12-DHAHSA (**B**) treatment. (**C**) The fluorescence intensity responses to oxidative stress induced by H_2_O_2_ and the effect of 12-DHAHOA treatment. * *p* < 0.05, *** *p* < 0.001, ns: not significant (one-way ANOVA) (*n* = 6, mean ± SEM).

The cytotoxicity of DHA, 12-HSA, 12-DHAHSA, and 12-DHAHOA was evaluated against cultured C3A cells by CCK-8 assay and the values are found to be 124.5 µM, 161 µM, 252 µM, and 225 µM, respectively. The percentages of cell viability logarithmic plots for the compounds are shown in Figure 2. However, cytotoxicity of 12-HOA is not evaluated in the current study but it should be expected to be similar to 12-HSA. The results show that DHA-derived FAHFAs are almost two-folds less toxic when compared to their respective free fatty acids. The ability of each DHA-derived FAHFAs towards the activation of NRF2 was examined with a reporter gene assay using the Dual-Glo Luciferase Reporter Assay System (Promega) by the protocols established in our lab [4,15]. In this assay, the antioxidant response element (ARE) was applied and drove the transcription of the luciferase reporter gene. If the NRF2 is activated, then it translocated to the nucleus where it binds to ARE protein and activates antioxidant signaling. Hence, higher luciferase activity refers to the strong activation of NRF2. The results of the reporter gene assay are shown in Figure 3A,B. These data suggest the dose-dependent activation of NRF2 by both 12-DHAHOA and 12-DHAHSA. However, the 12-DHAHOA seems to have a more potent activator of NRF2 compared to 12-DHAHSA. Furthermore, cellular reactive oxygen species (ROS) levels were determined by the protocol established earlier in our lab with minor modifications [15]. The oxidative stress in C3A cells was induced by treatment with the known concentration of hydrogen peroxide (0.25 mM) and the effect of 12-DHAHOA on ROS levels was measured. These data are shown in Figure 3C The results show a significant decrease in ROS levels at 125 µM treatment of 12-DHAHOA compared to the hydrogen peroxide treated group.

**Figure 4:**
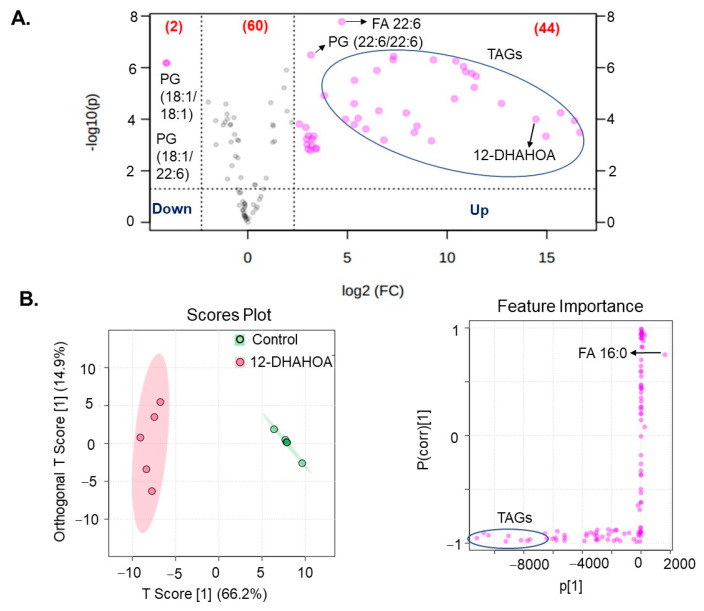
(**A**) Volcanic plot of all the annotated lipids (only species changed by 5-fold are highlighted with applied *t*-test of *p* < 0.05). (**B**) Orthogonal partial least squares discriminant analysis (OPLS-DA). Score plot and feature importance; colored circles display 95% confidence regions; Control (*n* = 5) and 12-DHAHOA (*n* = 5) (Predictive component 1: R2X = 0.662, R2Y = 0.965, Q2 = 0.954; Orthogonal component 1: R2X = 0.149 R2Y = 0.00218, Q2 = 0.0162; All components: R2X(cum): 0.811). Q2 and R2 values represent the predictability and goodness of fit, respectively.

Next, in order to investigate the effect of DHA-derived FAHFAs on cellular lipid pathways, the cells were treated with the known concentration of 12-DHAHOA (77 µM) and incubated at 37 °C for 24 h. After that, cells were extracted for total lipids and untargeted LC/MS analysis was performed to know the relative levels of multiple lipid classes. In total, about 106 lipids were identified and annotated based on MS/MS fragmentation behavior. The volcanic plot (Figure 4A) shows the significantly altered lipids between control and 12-DHAHOA treated groups. Out of 106, about 44 lipids are significantly increased by 5-fold from the control group. In contrast, only two phosphoglycerols (PG) (PG (18:1/18:1) and PG (18:1/22:6)) were shown to be decreased in the 12-DHAHOA treated group compared to the control. Among significantly increased lipids, triacylglycerols (TAGs) and FA 22:6 derived lipids are the major species. The list of all the significantly altered lipids was provided in Supplementary Materials Table S1. Furthermore, multivariate analysis such as OPLS-DA was performed to find the separation between the groups based on the most significant variables. The OPLS-DA score plot (Figure 4B) shows the clear separation between the control and 12-DHAHOA treated groups. The feature importance box plot (also known as S-Plot) (Figure 4B) visualizes the variable influence in an Orthogonal PLS-DA model. It combines covariance and correlation loading profiles. This corresponds to combining the contribution with the effect and reliability for the model variables with respect to the model component scores. The larger positive or negative loading scores indicate that a variable has a strong effect on the orthogonal components. In our results, TAGs showed a large negative loading, whereas phospholipids such as PG, Phosphoethanolamine (PE), Phosphatidylinositol (PI), and FA 16:0 showed large positive loadings. The list of lipid metabolites with their loading scores was provided in Supplementary Materials Table S2.

**Figure 5:**
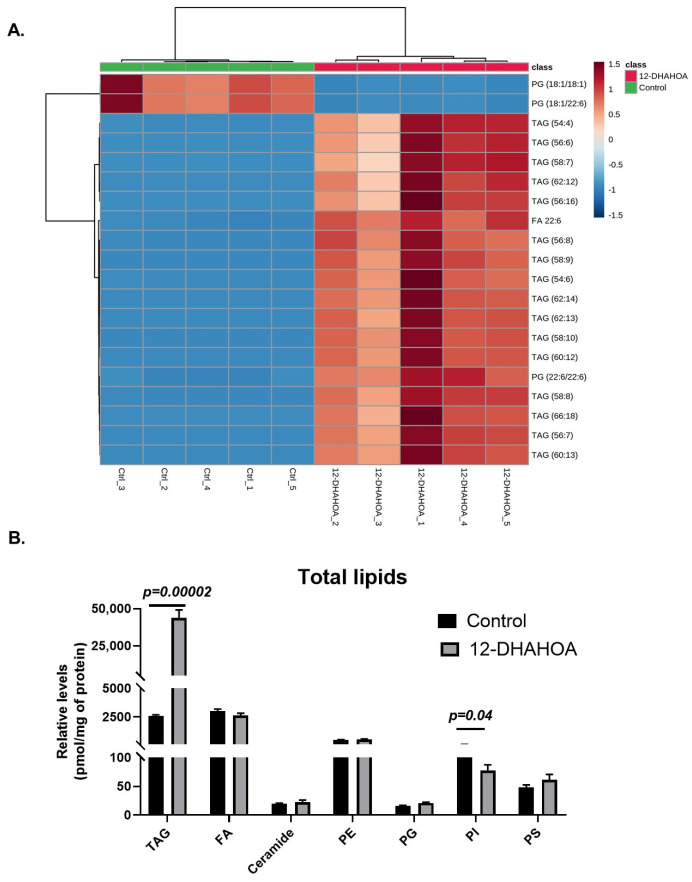
(**A**) Hierarchical cluster correlation analysis of top 20 significantly altered species (*t*-test, *p* < 0.05, distance measure: Euclidean; clustering algorithm: Ward. Each colored cell on the map corresponds to a concentration value of lipids, with samples in rows and compounds in columns. Red: increased, Blue: decreased). (**B**) Relative levels of total lipids in control (C3A) and 12-DHAHSA treated samples (*t*-test, *p* < 0.05) (mean ± SEM (*n* = 5). (TAG: Triacylglycerol, FA: Fatty acid, PE: Phosphatidylethanolamine, PG: Phosphatidylglycerol, PI: Phosphatidylinositol, and PS: Phosphatidylserine).

The multivariate hierarchical cluster correlation analysis was performed to identify the species that increased or decreased and the results of the top 20 significantly altered lipids were shown in Figure 5A. As the results demonstrate, the mainly TAGs with polyunsaturated fatty acid (especially FA 22:6) were increased significantly in 12-DHAHOA treated group compared to the control, whereas PG (18:1/18:1) and PG (18:1/22:6) showed the decreasing trend in 12-DHAHOA treated cells. Moreover, a tight clustering is observed between the control and 12-DHAHOA groups. Figure 5B shows the total relative level of each lipid class. This data also shows a significant increase in total TAGs by almost 17-fold in 12-DHAHOA treated samples compare to control. However, no significant changes were observed in other lipid classes except PIs, which shows a slight decrease in 12-DHAHOA treated group.

**Figure 6:**
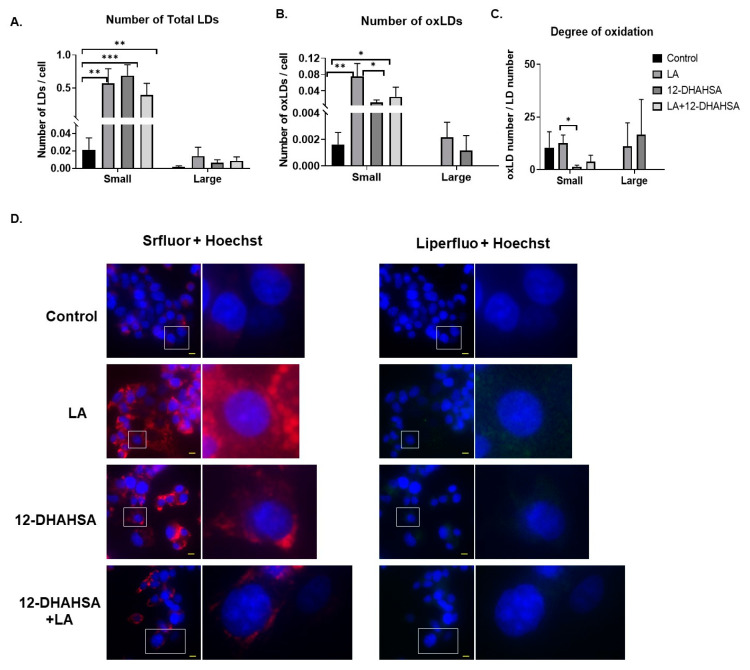
Effect of 12-DHAHSA (125 µM) on oxidation of LA-induced LDs in C3A cells. (**A**) Number of total LDs. (**B**) Number of oxLDs. (**C**) Degree of oxidation. Small LDs are < 3 µm^2^ and large LDs are ≥ 3 µm^2^ and are quantified by analyzing fluorescent images with ImageJ software. (**D**) Fluorescent images showed neutral lipid (red), lipid peroxide (green), and nuclei (blue). The scale bar shown in each image is 10 µm. Columns and bars represent the mean ± SEM (*n* = 3). Student’s *t*-test with * *p* < 0.1, ** *p* < 0.05, *** *p* < 0.01.

Furthermore, the antioxidant potential of 12-DHAHSA was evaluated by the fluorescent imaging technique. The 12-DHAHSA was selected because of its lower cytotoxicity compared to 12-DHAHOA. The lipid droplets (LDs) were induced by treatment of C3A cells with linoleic acid (LA) for 8 h and the effects of 12-DHAHSA on total and oxidized lipid droplets (oxLDs) were determined by the method established earlier in our laboratory [16]. There is a significant increase in the total number of LDs observed between the control and LA or 12-DHAHSA treated group (Figure 6A). However, a slightly decreasing trend is observed in total LDs with LA and the 12-DHAHSA treated group. Furthermore, the number of small oxLDs decreased with 12-DHAHSA treatment compared to LA treated group (Figure 6B). However, no significant changes were observed for large oxLDs. The degree of oxidation in small LDs was also decreased with 12-DHAHSA treatment. The fluorescent images showed the effect of 12-DHAHSA on LDs (SRfluor + Hoechst) and oxLDs (Liperfluo + Hoechst) are shown in Figure 6C.

## 3. Discussion

The development of NRF2 activators is a promising therapeutic tool for the treatment of oxidative stress-associated human diseases. NRF2 is a transcription factor that regulates the gene expression of antioxidant signaling enzymes. We have previously reported both natural and synthetic molecules such as Eicosapentaenoic acid esters of hydroxy fatty acids as potent NRF2 activators [4]. In enduring our FAHFA research, in this study, we explored DHA-derived FAHFAs as potent NRF2 activators. FAHFAs are recently discovered as endogenous lipid metabolites with multiple bioactivities such as anti-inflammatory [2], anti-diabetic [2,11], anti-apoptotic activities [3], and also promote browning of white adipose tissue [17]. Despite their structural diversity with more than 300 regioisomers [5], to date, the PAHSA family is the most studied [1,5,8]. The studies focusing on the effects of polyunsaturated FAHFAs such as ω-3 (DHALA and EPAHSA) and ω-6 (LAHLA) fatty acids are limited. Specifically, 13-DHAHLA and its enantiomeric forms have exhibited higher anti-inflammatory properties than 9-PAHSA [11]. Herein we synthesized two derivatives of DHA, i.e., 12-DHAHSA and 12-DHAHOA and examined their cytotoxicity against C3A cells. As the results showed, Figure 2 clearly demonstrates that the DHA-derived FAHFAs are almost 2-fold less cytotoxic than their native fatty acids, suggesting that they are potential candidates to screen for NRF2 activation. These results are similar to our previous study that demonstrated the lower cytotoxicity of EPA-derived FAHFAs compared to free fatty acids [4]. Further reporter gene assay results revealed that 12-DHAHOA and 12-DHAHSA as NRF2 activators, with the former being more potent at 125 µM and 250 µM, respectively. A past study demonstrated the protective effects of EPA and DHA in astrocytes against oxidative stress via NRF2-dependent signaling [18]. We also tested the activity of DHA itself by reporter gene assay and found a substantial increase in luciferase activity (Figure S1). Although DHA showed a higher affinity to increase the Nrf2 levels at a low concentration, it exhibits higher toxicity compared to DHA-derived FAHFAs. The direct antioxidant effect of 12-DHAHOA was evaluated by inducing oxidative stress in C3A cells using H_2_O_2_. The results showed the decreasing tendency in oxidative-stress-induced reactive oxygen species (ROS) levels with treatment of 125 µM 12-DHAHOA (Figure 2), which supports DHA-derived FAHFA as a plausible antioxidant. Again, these results are consistent with EPA-derived FAHFAs, which reduced ROS levels significantly in HepG2 cells [4].

Liquid chromatography/mass spectrometry is the widely applied technique for measuring endogenous lipids, including FAHFAs [14,19]. In order to reveal the lipid pathway, which is altered by the 12-DHAHOA treatment, we performed an untargeted lipidomic analysis. Our results suggested a significant difference in the multiple lipid molecular species between the control and 12-DHAHOA treated groups. The most interesting fact is about 44 molecular species were significantly upregulated by 5-fold in the 12-DHAHOA group, with the majority of TAGs. The OPL-SDA score plots also suggested the distinct lipid profiles between 12-DHAHOA treated and untreated groups. TAGs are the top molecular species upregulated with 12-DHAHOA treatment, which is demonstrated by the hierarchical cluster correlation heatmap (Figure 5A). Additionally, total TAG levels are also increased 17-fold suggesting the strong effect of 12-DHAHOA on TAG metabolism. The involvement of NRF2 in regulation of lipid metabolism is extensively reviewed by He F et al. and discussed both NRF2 dependent and independent triglyceride production [20]. For instance, NRF2 activation in hepatocytes induced triglyceride accumulation in liver [21] and these results are consistent with our study. We assume that activation of NRF2 by 12-DHAHOA could be the cause for increased TAGs in C3A cells. Moreover, previous studies have also demonstrated the abundance of FAFHFA-TAGs over white adipose tissue and are believed to be an important reservoir of FAHFAs in cells and tissues [5]. They also reported that FAHFAs are incorporated into TAGs by the action of diacylglycerol acyltransferases DGAT1 and DGAT2 in adipocytes in vitro [22]. However, in our study, we have not detected any FAHFA-TAGs. This is probably due to a technical limitation or the lack of expression of DGATs in C3A cells, which needs further investigation. There is no change in other lipid metabolism observed with the 12-DHAHOA treatment except TAGs and PIs. Total PIs are reduced relatively compared to the control group. As per our knowledge, this is the first report to show the impact of FAHFAs on PIs biosynthesis. Moreover, the increase in FA 22:6 containing TAGs is probably due to the incorporation of DHA from the 12-DHAHOA. However, to confirm this hypothesis, isotope labelling studies are required, which limits this study. Furthermore, LDs specific fluorescent imaging analysis revealed that 12-DHAHSA possesses similar antioxidant effects to that of 12-EPAHSA [4]. It reduced the small oxLDs significantly and suggested a protective role of DHA-derived FAHFAs under oxidative stress damages. Hence, further investigations are necessary to reveal the in vivo effects of these novel FAHFAs and their mechanism of action.

## 4. Materials and Methods

### 4.1. Materials

The solvents such as methanol, isopropanol, chloroform, ammonium acetate solution (1M) of LC/MS grade, Dicyclohexyl carbodiimide, and lithium hydroxide were obtained from Wako Pure Chemical Industries, Ltd., (Osaka, Japan). 12-hydroxystearic acid, methyl ricinoleate, dry pyridine, 4-dimethylaminopyridine, and anhydrous dichloromethane were purchased from the Tokyo Chemical Industry (Tokyo, Japan). Docosahexaenoic acid with a purity ≥98% was obtained from Cayman Chemicals (Ann Arbor, MA, USA). Silica Gel N60 (40–50 μm) was obtained from Kanto Chemical Industry (Tokyo, Japan). The EquiSPLASH Lipidomix quantitative standard for mass spectrometry and oleic acid-d9 internal standards were purchased from Avanti Polar Lipids (Alabaster, AL, USA). Silica gel 60G F_254_ glass plates (20 × 20 cm^2^) were obtained from Merck (Tokyo, Japan). The high-resolution mass measurements were performed by Linear Quadrupole Orbitrap mass spectrometer (LTQ Orbitrap XL, Thermo Fisher Scientific (San Jose, CA, USA)).

### 4.2. Methods

#### 4.2.1. Characterization of DHA-Derived FAHFAs

The standards such as 12-DHAHSA and 12-DHAHOA were prepared according to the protocols reported in our earlier study [4]. After chromatographic column purifications, the standards were subjected to LC/MS analysis using Prominence UHPLC system (Shimadzu Corp., Kyoto, Japan) connected to an LTQ Orbitrap MS (Thermo-Fisher Scientific Inc., San Jose, CA, USA). The MS analysis was carried out in ESI-negative ionization mode. The analysis was carried out according to the conditions reported in our earlier study [14].

#### 4.2.2. Cell Culture

Human hepatoma-derived cells (C3A, CRL-10741) were obtained from American Type Culture Collection (Manassas, VA, USA) and stored at −80 °C. The cells were kept in a minimum essential medium (MEM, Thermo Fisher Scientific (Tokyo, Japan)) augmented with 10% (*v*/*v*) fetal bovine serum (FBS, Thermo Fisher Scientific (Tokyo, Japan)) and an 1% (*v*/*v*) antibiotic mixture of Penicillin-Streptomycin (PS, Wako Pure Chemicals) at 37 °C under a humidified atmosphere of 5% CO_2_ in air. The MEM having FBS and PS is here on refered to as modified MEM.

#### 4.2.3. Cytotoxicity Assay

C3A cells (1.2 × 10^5^/well) were seeded into 96-well plates with modified MEM and incubated at 37 °C for 24 h. Then, the media were removed and the compounds (12-DHAHSA, 12-DHAHSA) were pre-dissolved in dimethyl sulfoxide (DMSO) and diluted with MEM (with final concentration to 0.1% DMSO) and then separately applied to the cells and further maintained at 37 °C for 24 h. The compounds containing the media were then removed, 200 μL of MEM and 10 μL of CCK-8 reagent (Dojindo Molecular Technologies, Kumamoto, Japan) were added and the plates were incubated at 37 °C for 1 h. The reaction was terminated by adding 50 μL of 1% (*w*/*v*) sodium dodecyl sulfate solution and the absorbance was measured at 450 nm with a Wallac 1420 ARVO Mx plate reader (PerkinElmer, Tokyo, Japan). The half-maximal inhibitory concentration (IC50) of 12-DHAHSA and 12-DHAHOA was analyzed using a non-linear transformation with Prism 8.01 (GraphPad, San Diego, CA, USA) software. All procedures for CCK-8 were performed according to a previously described protocol with minor modifications [4,15].

#### 4.2.4. Reactive Oxygen Species (ROS)

The cellular oxidative stress due to H_2_O_2_-induced ROS was measured by the dichlorofluorescein-diacetate (DCFH-DA) assay with the protocol established in our laboratory [4,15]. A condition of cellular stress in C3A cells is evoked by treating 0.25 mM H_2_O_2_ for 24 h and fluorescence intensity was read at 485 (excitation) and 525 (emission) nm using Wallac 1420 ARVO Mx plate reader (PerkinElmer, Tokyo, Japan).

#### 4.2.5. Reporter Gene Assay to Determine NRF2 Activation

Approximately 1.2 × 10^5^/well C3A cells were seeded into 96-well plates with modified MEM and incubated at 37 °C for 24 h. Transfections were carried out using the FuGENE HD Transfection Reagent, according to the manufacture’s protocol. The reagent-to-total DNA ratio was kept at 4:1 (μL/μg). Luciferase reporter vectors, pGL4.37[luc2p/ARE/Hygro] and pGL4.75[hRluc/CMV] (Promega, Tokyo, Japan) were used at a 20:1 mass ratio for transfection of C3A cells. The vector pGL4.37[luc2p/ARE/Hygro] contains four copies of an antioxidant responsive element (ARE) and the luciferase reporter gene luc2P (Photinus pyralis). The vector pGL4.75[hRluc/CMV] contains the luciferase reporter gene hRluc (Renilla reniformis). After 24 h of transfection, the transfection reagent/DNA mixture was removed. The 12-DHAHOA and 12-DHAHSA pre-dissolved in DMSO and diluted in MEM were separately applied to the transfected cells and further incubated for 24 h. Then, the luciferase activity was determined using the Dual-Glo Luciferase Assay System (Promega, Tokyo, Japan) and activity was measured with a Wallac 1420 ARVO Mx plate reader by the protocol established earlier in our lab [4,15]. The obtained data are adjusted for transfection efficiency by normalizing to hRluc activity. The fold change in the relative activity of luciferase was calculated by taking the ratio of fluorescence intensity of the treated and untreated sample.

#### 4.2.6. Total Lipid Extraction and Analysis

Equal number of C3A cells were seeded into 6-well plates with 2 mL of modified MEM and incubated at 37 °C for 24 h. After that, the media was removed, cells were treated with 0.1% DMSO (control) and 77 µM of 12-DHAHOA and further incubated at 37 °C for 24 h. The media were removed and the cells were washed with PBS. The total lipids were extracted by the Folch method with modifications as established earlier in our laboratory [23,24]. In brief, cells were homogenized in 100 µL methanol for the 30 s (x 2 cycles) using the Bead Mill 4 (Fisherbrand, Tokyo, Japan) Homogenizer. Then, 100 µL internal standard mixture in methanol (340 µM of oleic acid (d9), 13.2 µM of phosphatidylcholine (PC) (15:0-18:1(d7)), 14 µM of phosphatidylethanolamine (PE) (15:0-18:1(d7)), 12.7 µM of phosphatidylglycerol (PG) (15:0-18:1(d7)), 12.5 µM of phosphatidylserine (PS) (15:0-18:1(d7)), 11.5 µM of phosphatidylinositol (PI) (15:0-18:1(d7)), 20.5 µM of lysophosphatidylethanolamine (LPE) (18:1(d7)), 18.9 µM of lysophosphatidylcholine (LPC) (18:1(d7)), 13.5 µM of sphingomyelin (SM) (d18:1/18:0(d9)), 18.8 µM of ceramide (Cer) (d18:1/15:0 (d7)), 12.3 µM of triacylglycerol (TAG) (15:0-18:1(d7)-15:0), 17 µM of diacylglycerol (DAG) (15:0-18:1(d7)), 15.1 µM of cholesterol ester (18:1(d7)), and 27.5 µM of monoacylglycerol (18:1(d7)) were added and vortexed at 3500 rpm for the 30 s. Followed by 400 µL of chloroform and 100 µL of milli-Q were added and vortexed for 5 min. The biphasic extracts were centrifuged (15,000 rpm, 10 min at 4 °C) and the chloroform layer was transferred to a new vial. The water layer was re-extracted with an additional 400 µL of chloroform. The unified chloroform extracts were evaporated and 100 µL of methanol was added to redissolve the lipid residue. About 10 µL of the sample was injected into the LC/MS.

The analysis was performed on the prominence UHPLC system (Shimadzu Corp., Kyoto, Japan) coupled to an LTQ Orbitrap mass spectrometer (Thermo-Fisher Scientific Inc., San Jose, CA, USA). The chromatographic separation was achieved using a reverse-phase Atlantic T3 C18 column (2.1 × 150 mm, 3 µm, Waters, Milford, MA, USA) at an oven temperature of 40 °C. The separation method, MS parameters (both positive and negative mode), and annotation of lipids are identical to that of our previous reports [23,24]. The high-resolution MS data were acquired in Fourier transform mode with a resolving power of 60,000 and a collision energy of 35 eV, with a scan range *m*/*z* 160–1900. Data-dependent acquisitions with MS/MS were performed in the ion-trap mode at a precursor ion isolation width of 3 *m*/*z* units and a collision energy of 40 eV. All the annotated lipids are confirmed by their MS/MS spectra and retention time behavior with that of the internal standard. Extracted ion chromatograms and MS spectra were obtained using Xcalibur 2.2 (Thermo-Fisher Scientific Inc.). The quantification is carried out after integrating the peaks using Xcalibur 2.2 (Thermo-Fisher Scientific Inc., San Jose, CA, USA) software, according to the guidelines of Lipidomics Standards Initiative level 2 and level 3 (https://lipidomics-standards-initiative.org/). The relative levels after normalizing the peak area by protein content are calculated by taking the analyte’s peak area ratios to the internal standard and multiplying it using the added internal standard.

#### 4.2.7. Protein Determination

C3A cells were cultured by a method similar to that used for total lipid extraction. The cell pellet was prepared by centrifuging at 3500 rpm for 10 min at 4 °C. To the cell pellet in 1.5 mL Eppendorf, 0.5 mL of milli-Q and 0.5 mL of 0.1 mM NaOH were added, gently vortexed, and kept in a thermostat at 60 °C for 2 h. About 25 µL was transferred into a 96-well plate (*n* = 4) and 200 μL of a reaction reagent (50:1 mixture of reagent A and reagent B) was added (Bicinchoninic protein assay kit, Thermo Fisher SCIENTIFIC, Tokyo, Japan). Furthermore, it was incubated at 37 °C for 30 min and absorbance was measured at 562 nm with a Wallac 1420 ARVO Mx plate reader. Bovine serum albumin (BSA) (2000 µg/mL) from the Thermo Fisher SCIENTIFIC (Tokyo, Japan) was used to prepare the calibration curve by serial dilutions to 2000, 1000, 100, 10, 1, and 0.1 µg/mL with milli-Q. The absorbance of the standard was also recorded after performing a similar protocol as above.

#### 4.2.8. Fluorescent Imaging

The fluorescent imaging of lipid droplet (LD) and oxidized lipid droplet (oxLD) were carried according to the protocol established in our earlier study using a BZ-9000 fluorescence microscope (Keyence Co. Ltd., Osaka, Japan) [16]. Briefly, 2 × 10^5^ cells were precultured in a 0.1% gelatin-coated glass-bottom dish with 2 mL of the modified MEM at 37 °C for 24 h. Then, the medium was removed and the cells were treated with 400 µM linoleic acid (LA) with 125 µM 12-DHAHSA. After incubation at 37 °C for 8 h, the cells were stained for 30 min at 37 °C using a neutral lipid specific fluorescent probe (5 µM SRfluor 680-phenyl, Funakoshi co. Ltd., Tokyo, Japan) and lipid peroxides specific fluorescent probe (10 µM Liperfluo and 10 µg/mL Hoechst33342, Dojindo Laboratories, Kumamoto, Japan). Fluorescence was recorded with the following filter sets: excitation: 360/40 nm, emission: 460/50 nm, dichroic mirror: 400 nm (blue); excitation: 470/40 nm, emission: 525/50 nm, dichroic mirror: 495 nm (green); excitation: 620/60 nm, emission: 700/75 nm, dichroic mirror: 660 nm (red). Images were analyzed by ImageJ 1.50i software. oxLD-positive images were obtained by binarizing the bright field, SRfluor, and Liperfluo images and obtaining the intersectional images of each. The non-oxLD image was obtained by inverting only the binarized Liperfluo image and then crossing the binarized bright field and SRfluor. The total number of LDs was calculated as the total of oxLD and non-oxLD.

#### 4.2.9. Statistical Analysis

Statistical analyses were conducted using the GraphPad Prism 8.1 software. Student’s *t*-test (*p* < 0.05) or one-way ANOVA (* *p* < 0.05, ** *p* < 0.01, *** *p* < 0.001, ns: not significant) were used to study statistically significant differences between the groups. The multivariate analysis (OPLS-DA and Hierarchical clustering analysis) was performed by MetaboAnalyst 5.0.

## 5. Conclusions

In conclusion, this in vitro study demonstrated that DHA-derived FAHFAs are a novel class of lipids with less cytotoxic and potent NRF2 activators in human hepatoma cells. Moreover, it reduced the oxidative stress induced by H_2_O_2_ and decreased the number of oxLDs, strongly suggesting their plausible antioxidant action. The lipidomic analysis revealed the significant upregulation of TAG metabolism with FAHFAs treatment. Further investigations are necessary to reveal the molecular mechanisms behind NRF2 activation and altered lipid metabolism.

## Data Availability

The data are available upon request to first or corresponding author.

## Supplementary Materials

The followings are available online at https://www.mdpi.com/article/10.3390/ijms22147598/s1.
