# Supplementary material for: Docosahexaenoic Acid Esters of Hydroxy Fatty Acid Is a Novel Activator of NRF2
Source: Int J Mol Sci. 2021 Jul 15;22(14):7598. doi: 10.3390/ijms22147598 (PMC8306801; doi:10.3390/ijms22147598)

Table S1: The list of significantly altered lipids with >5 folds change.

Lipid	FC	log2(FC)	raw.pval	LOG10(p)
FA 22:6	26.241	4.7137	1.65E-08	7.7812
PG (22:6/22:6)	8.9811	3.1669	3.22E-07	6.4926
TAG (56:8)	158.79	7.3109	3.67E-07	6.435
TAG (58:8)	156.16	7.2869	4.94E-07	6.3063
TAG (58:9)	635.42	9.3116	5.00E-07	6.3009
TAG (58:10)	1387.1	10.438	5.56E-07	6.2549
PG (18:1/18:1)	0.059104	-4.0806	6.51E-07	6.1862
PG (18:1/22:6)	0.059127	-4.08	6.67E-07	6.1762
TAG (60:12)	1811.4	10.823	9.07E-07	6.0425
TAG (56:7)	88.974	6.4753	1.28E-06	5.8912
TAG (62:13)	1959.5	10.936	1.44E-06	5.8414
TAG (62:14)	2415	11.238	1.67E-06	5.7775
TAG (60:13)	2807	11.455	2.15E-06	5.6667
TAG (54:6)	40.752	5.3488	3.10E-06	5.5085
TAG (66:18)	2629.9	11.361	5.82E-06	5.235
TAG (54:4)	14.244	3.8323	1.21E-05	4.9175
TAG (62:12)	1320.6	10.367	1.60E-05	4.7961
TAG (56:16)	6789.1	12.729	2.44E-05	4.6125
TAG (56:6)	40.322	5.3335	2.50E-05	4.6028
TAG (58:7)	95.44	6.5765	4.72E-05	4.3264
TAG (62:15)	52887	15.691	5.64E-05	4.2491
TAG (60:8)	246.71	7.9467	5.74E-05	4.2413
CL (68:2)	46.307	5.5332	9.18E-05	4.0374
12DHAHOA	22387	14.45	9.98E-05	4.0009
CL (64:0)	29.952	4.9046	0.000101	3.9976
TAG (64:17)	85901	16.39	0.000113	3.9474
TAG (52:2)	6.0159	2.5888	0.000153	3.8147
TAG (58:6)	40.42	5.337	0.000162	3.7909
CL (76:12)	358.71	8.4867	0.000184	3.7361
TAG (54:3)	7.5426	2.9151	0.00021	3.6777
TAG (60:7)	60.754	5.9249	0.00024	3.6194
TAG (58:13)	103200	16.655	0.000324	3.4896
TAG (62:8)	326.68	8.3517	0.000327	3.4857
TAG (53:2)	8.2242	3.0399	0.000434	3.3628
TAG (52:1)	9.9042	3.308	0.000454	3.3428
TAG (56:12)	31812	14.957	0.000457	3.3406
TAG (55:3)	7.7314	2.9507	0.000585	3.233
TAG (54:2)	9.3769	3.2291	0.000585	3.233
TAG (62:7)	113.2	6.8227	0.000648	3.1887
TAG (64:8)	594.63	9.2158	0.000695	3.1583
TAG (56:3)	7.866	2.9756	0.000954	3.0206
TAG (54:1)	9.2226	3.2052	0.00102	2.9915
TAG (58:2)	10.901	3.4463	0.001313	2.8817
TAG (56:2)	8.1229	3.022	0.00144	2.8416
TAG (60:3)	10.615	3.408	0.001455	2.8371
TAG (58:3)	8.8447	3.1448	0.001643	2.7844

Table S2: The list of lipid metabolites with their loading scores corresponds to OPLSDA plot.

Lipid	p[1]	p(corr)[1]	Lipid	p[1]	p(corr)[1]
PG (18:1/18:1)	22.67	0.99	TAG (52:1)	-6558.30	-0.91
PG (18:1/22:6)	22.66	0.99	TAG (56:12)	-3714.80	-0.91
PE (18:1/22:5)	49.73	0.97	TAG (53:2)	-1136.60	-0.91
PI (18:0/20:2)	31.64	0.97	12-DHAHOA	-121.46	-0.92
PI (18:1/20:4)	53.15	0.96	TAG (62:8)	-3178.00	-0.92
PE (16:0/20:4)	57.35	0.95	TAG (58:13)	-2205.40	-0.92
PE (16:0/18:1)	39.42	0.95	TAG (60:7)	-5222.10	-0.92
PI (16:1/18:1)	8.42	0.95	TAG (54:3)	-10409.00	-0.93
PE (18:1/20:4)	173.39	0.94	PE (18:1/22:6)	-176.55	-0.93
PI (18:0/18:1)	19.56	0.94	TAG (58:6)	-3730.80	-0.93
PI (16:0/20:4)	15.87	0.94	TAG (52:2)	-9051.80	-0.93
PI (18:0/22:4)	8.58	0.94	TAG (50:1)	-3010.40	-0.93
PE (18:0/20:4)	255.58	0.93	FA 18:3	-5.67	-0.93
PS (18:0/22:5)	34.25	0.91	TAG (64:17)	-2642.40	-0.94
PE (16:1/18:1)	40.00	0.91	TAG (62:15)	-5834.10	-0.95
PE (18:0/20:3)	13.36	0.90	TAG (60:8)	-5196.20	-0.95
PE (18:0/18:1)	32.96	0.90	TAG (58:7)	-11198.00	-0.95
LPE-N (FA 18:1)	163.06	0.88	TAG (56:16)	-6438.50	-0.96
FA 18:1	79.47	0.82	TAG (56:6)	-6033.40	-0.96
PS (18:0/18:1)	47.91	0.82	TAG (62:12)	-3059.20	-0.96
FA 16:0	1633.10	0.75	PE (18:0/22:6)	-486.96	-0.96
TAG (58:3)	-2225.20	-0.87	TAG (52:3)	-1646.90	-0.96
TAG (56:2)	-2985.80	-0.88	TAG (66:18)	-7590.00	-0.97
TAG (60:3)	-1683.90	-0.88	TAG (54:6)	-2263.30	-0.97
TAG (58:2)	-1724.00	-0.88	TAG (54:4)	-2937.00	-0.97
CL (76:12)	-10.27	-0.89	TAG (62:14)	-8182.50	-0.97
TAG (54:1)	-1971.30	-0.89	TAG (60:13)	-3054.30	-0.97
TAG (56:3)	-5710.50	-0.89	TAG (62:13)	-5777.90	-0.97
PE (16:0/22:6)	-162.36	-0.90	TAG (60:12)	-5228.20	-0.98
FA 18:2	-37.70	-0.90			
CL (64:0)	-14.84	-0.90			
TAG (64:8)	-3068.20	-0.90			
CL (68:2)	-21.16	-0.90			
TAG (62:7)	-3419.80	-0.90			
TAG (54:2)	-10743.00	-0.90			
TAG (55:3)	-809.39	-0.90			

Figure S1*:* Reporter gene assay results of DHA. * p < 0.05, ** p < 0.01, *** p < 0.001, ns: not significant (one-way ANOVA) (n = 6).

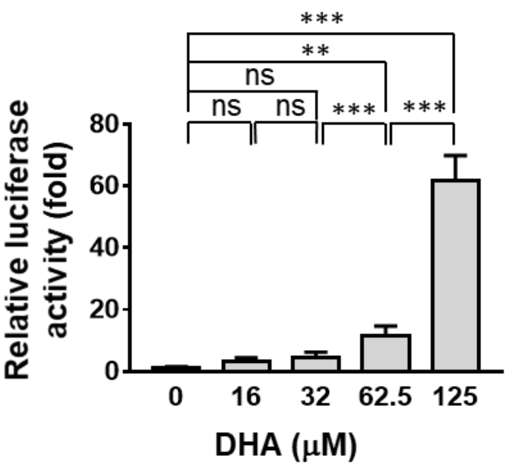

Supplement: Supplementary file 1 [file ijms-22-07598-s001.zip › ijms-1275762-supplementary.pdf]
